# The Effect of Disability and Social Determinants of Health on Breast and Cervical Cancer Screenings During the COVID-19 Pandemic

**DOI:** 10.5888/pcd21.230234

**Published:** 2024-01-25

**Authors:** LaShae D. Rolle, Maurice J. Chery, Michaela Larson, Melissa Lopez-Pentecost, Carmen J. Calfa, Matthew P. Schlumbrecht, Tracy E. Crane

**Affiliations:** 1 Department of Public Health Sciences, University of Miami Miller School of Medicine, Miami, Florida; 2 Sylvester Comprehensive Cancer Center, University of Miami Miller School of Medicine, Miami, Florida; 3 Department of Obstetrics, Gynecology, and Reproductive Sciences, Miller School of Medicine, Miami, Florida

## Abstract

**Introduction:**

The objective of this study was to examine the effect of disability status and social determinants of health (SDOH) on adherence to breast and cervical cancer screening recommendations during the COVID-19 pandemic.

**Methods:**

We conducted a secondary analysis of the 2018 and 2020 Behavioral Risk Factor Surveillance System (BRFSS) data sets. We defined adherence to screenings according to the US Preventive Services Task Force guidelines for breast and cervical cancer screening. The analysis included respondents assigned female at birth, aged 50 to 74 years (breast cancer screening) or aged 21 to 65 years (cervical cancer screening). We performed logistic regression to evaluate breast and cervical cancer screening adherence, by disability status and SDOH (health insurance coverage, marital status, and urban residency), independently and simultaneously.

**Results:**

Our analysis included 27,526 BRFSS respondents in 2018 and 2020. In 2018, women with disabilities had lower adjusted odds than women without disabilities of being up to date with mammograms (adjusted odds ratio [AOR] = 0.76, 95% CI, 0.63–0.93) and Pap (Papanicolaou) tests (AOR = 0.73; 95% CI, 0.59–0.89). In 2020, among women with disabilities, the adjusted odds of mammogram and Pap test adherence decreased (AOR = 0.69; 95% CI, 0.54–0.89; AOR = 0.59; 95% CI, 0.47–0.75, respectively). In 2018, the adjusted odds of mammogram adherence among rural residents with and without disabilities were 0.83 (95% CI, 0.70–0.98), which decreased to 0.76 (95% CI, 0.62–0.93) in 2020.

**Conclusion:**

The findings of this study highlight the effect of disability status and SDOH on breast and cervical cancer screening rates during the COVID-19 pandemic. Public health strategies that acknowledge and address these disparities are crucial in preparing for future public health crises.

**Summary**

**What is already known on this topic?**

Prior research investigated the effect of disability status and social determinants of health on cancer screenings. Few studies have considered the implications of these factors on breast and cervical cancer screening during health crises such as the COVID-19 pandemic.

**What is added by this report?**

We compared cancer screening rates among women before (2018) and amid (2020) the COVID-19 pandemic. Women with disabilities and lower income, and women lacking health insurance coverage had reduced odds of being up to date on mammograms and Pap tests, before as well as amid the COVID-19 pandemic.

**What are the implications for public health practice?**

The findings highlight the critical need for health policies and interventions tailored for people who have disabilities and are socially marginalized, especially during times of health crises, when disparities, including disparities in access to essential preventive screenings, are exacerbated.

## Introduction

Breast cancer is the most prevalent type of cancer among women in the US; an estimated 287,850 cases and 43,250 deaths attributed to breast cancer occur annually (1). Additionally, nearly 13,000 new cases of cervical cancer and 4,000 cervical cancer deaths occur annually (2). Adherence to cervical cancer screening recommendations can substantially mitigate the incidence and death associated with the disease. Similarly, biennial breast cancer screenings can decrease breast cancer mortality by up to 40% (3–5). However, disparities in breast and cervical cancer screening rates and access to health care services persist according to race, ethnicity, and social determinant of health (SDOH), and these disparities were exacerbated during the COVID-19 pandemic (6–9). In 2020, the pandemic led to a reduction or halt in breast and cervical cancer screening services in many parts of the US (7,8,10), and the precise implications arising from these reductions in cancer screening as a result of this global event are inconclusive.

Approximately 61 million adults in the US live with a disability (11). A disability is a condition that impairs normal body function or cognition, restricts activity, and limits participation in societal roles (11). The nature and effect of disabilities, which can be congenital, developmental, injury-related, or associated with other health conditions, are diverse and can affect areas such as vision, movement, thinking, communication, and social relationships (11). Cancer screening uptake among people with disabilities is lower than among people without disabilities (12). Disability status and SDOH can substantially affect breast and cervical cancer screening rates. People with disabilities, particularly those with low socioeconomic status, have lower rates of breast and cervical cancer screening (13). Addressing disparities in cancer screening uptake among people with disabilities and varying socioeconomic circumstances calls for a multilevel, comprehensive approach that goes beyond individual interventions to address the broader SDOH. Interventions, such as tailored education programs, can enhance awareness and understanding of the importance of regular screenings (14). The objective of this study was to fill gaps in knowledge by investigating disparities in adherence to breast and cervical cancer screening among women with disabilities; exploring the effect of SDOH, including health insurance coverage, income, marital status, employment, education, and urban residency, during the COVID-19 pandemic; and assessing the degree of need for tailored interventions to improve access and use of screening services and address health equity.

## Methods

We conducted a secondary analysis of data from the 2018 and 2020 Behavioral Risk Factor Surveillance System (BRFSS). BRFSS is an annual, nationwide cross-sectional survey that collects data on risk behaviors, chronic health conditions, and use of preventive services by US residents. In 2018, BRFSS had an overall landline response rate of 53.3% and a cell phone response rate of 43.4% (15), resulting in 437,436 records collected for 2018. In 2020, BRFSS had an overall response rate of 47.9% (16), collecting 401,958 records for the year. The inclusion criteria for our study were based on US Preventive Services Task Force (USPSTF) recommendations for breast cancer screening updated in 2016 (17) and recommendations for cervical cancer screening updated in 2018 (18). For breast cancer screening, our analysis included respondents aged 50 to 74 years assigned female at birth (hereinafter, women); we considered respondents who received a mammogram in the previous 2 years to be up to date with screening. For cervical cancer screening, our analysis included respondents aged 21 to 65 years assigned female at birth (hereinafter, women); we considered respondents who received a Papanicolaou (Pap) test in the previous 3 years to be up to date with screening. We used the weighted calculated variables procedures outlined by BRFSS and applied weight, cluster, and strata variables to obtain population-based estimates and odds ratios (ORs) representative of the general population of US women (19).

### Dependent variables

The BRFSS-calculated variables MAM5023 (women aged 50–74 years who had a mammogram in the previous 2 years) and _RFPAP35 (women aged 21–65 years who had a Pap test in the previous 3 years) were the main dependent variables.

### Independent variables

Per the guidelines from the Centers for Disease Control and Prevention’s “A Data Users’ Guide to the Disability Questions,” we combined the variables deaf; blind; difficulty concentrating, remembering, or making decisions; difficulty walking or climbing stairs; difficulty dressing or bathing; and difficulty doing errands alone due to a physical, mental, or emotional condition to create the binary (yes/no) variable disability (20). We included race and ethnicity to investigate the intersection of race and ethnicity and screening in the sample. We included the variables health insurance coverage, annual household income, marital status, employment, educational attainment, and urban or rural residence in the multivariate regression models. These variables represent key SDOH, in alignment with the Healthy People 2030 SDOH domains: economic stability (income), education access and quality (educational attainment), health care access and quality (health insurance coverage), neighborhood and built environment (urban or rural residence), and social and community context (marital status).

### Statistical analyses

We first conducted descriptive analyses to characterize the sample of women, categorizing them as either up to date or not on mammograms and Pap tests, by disability status and SDOH. We generated bivariate and multivariable logistic regression models to examine the association between disability status and SDOH (independently and simultaneously) and the odds of being up to date on mammograms and Pap tests. We evaluated SDOH through measures of health insurance coverage, annual household income, marital status, employment, educational attainment, and urban or rural residence. We assessed the odds of women with disabilities being up to date on mammograms and Pap tests, taking into account the influence of SDOH by using a domain statement. All tests were 2-sided, with an α of < .05. We used SAS version 9.4 (SAS Institute, Inc) for all statistical analyses.

## Results

Of the 27,526 respondents in both years, a substantial majority were current with mammograms and Pap tests in both 2018 and 2020. In 2018, 78.4% (n = 13,138) were up to date on mammograms, and 78.4% (n = 13,067) were up to date on Pap tests. In 2020, 77.8% (n = 8,388) were up to date on mammograms and 77.4% (n = 8,235) on Pap tests. In 2018, 24.6% (4,099 of 16,669) of respondents reported having a disability; in 2020, 22.6% (n = 2,456 of 10,857) of respondents reported having a disability. Among women with disabilities, 72.1% (n = 2,991) were up to date on mammograms in 2018, and 69.6% (n = 1,744) were up to date in 2020. Pap test uptake among women with disabilities was 69.4% (n = 2,915) in 2018 and 66.1% (n = 1,639) in 2020 (Table 1). 

**Table 1:** Sample Characteristics by Mammogram and Pap Test Uptake in 2018 (n = 16,669) and 2020 (n = 10,857), BRFSS

Variable	Up to date on mammogram, no. (%^a^)				Up to date on Pap test, no. (%^a^)			
	2018		2020		2018		2020	
	Yes	No	Yes	No	Yes	No	Yes	No
**Overall**	13,138 (78.4)	3,531 (21.6)	8,388 (77.8)	2,469 (22.2)	13,067 (78.4)	3,602 (21.6)	8,235 (77.4)	2,622 (22.6)
**Disability**								
Yes	2,991 (72.1)	1,108 (27.9)	1,744 (69.6)	712 (30.4)	2,915 (69.4)	1,184 (30.6)	1,639 (66.1)	817 (33.9)
No	10,147 (80.7)	2,423 (19.3)	6,644 (80.4)	1,757 (19.6)	10,152 (81.5)	2,418 (18.5)	6,596 (81.0)	1,805 (19.0)
**Health insurance coverage**								
Yes	12,715 (96.1)	3,101 (85.6)	8,131 (96.6)	2,150 (85.9)	12,595 (95.7)	3,221 (87.1)	7,939 (95.9)	2,342 (88.3)
No	423 (3.9)	430 (14.4)	257 (3.4)	319 (14.1)	472 (4.3)	381 (12.9)	296 (4.1)	280 (11.7)
**Annual household income, $**								
<25000	2,271 (17.9)	1,008 (29.1)	1,316 (15.8)	656 (28.6)	2,224 (17.2)	1,055 (31.4)	1,254 (15.1)	718 (30.6)
25,000–49,999	2,575 (16.9)	891 (24.3)	1,656 (19.7)	604 (20.8)	2,537 (17.1)	929 (23.5)	1,595 (19.5)	665 (21.5)
50,000–74,999	2,422 (17.5)	576 (15.3)	1,489 (15.9)	433 (16.4)	2,400 (17.6)	598 (14.8)	1,468 (16.0)	454 (15.9)
>75,000	5,870 (47.7)	1,056 (31.3)	3,927 (48.6)	776 (34.2)	5,906 (48.0)	1,020 (30.2)	3,918 (49.3)	785 (32.0)
**Marital status**								
Married	8,185 (69.6)	1,888 (60.9)	5,396 (70.0)	1,381 (60.5)	8,213 (70.0)	1,860 (59.8)	5,373 (70.2)	1,404 (59.8)
Not married	4,953 (30.4)	1,643 (39.1)	2,992 (30.0)	1,088 (39.5)	4,854 (30.0)	1,742 (40.2)	2,862 (29.8)	1,218 (40.2)
**Employment**								
Employed	8,002 (58.6)	1,997 (54.3)	5,051 (57.5)	1,352 (51.2)	8,099 (60.5)	1,900 (47.4)	5,022 (57.8)	1,381 (50.2)
Unemployed	5,136 (41.4)	1,534 (45.7)	3,337 (42.5)	1,117 (48.8)	4,968 (39.5)	1,702 (52.6)	3,213 (42.2)	1,241 (49.8)
**Education**								
Some high school	403 (6.4)	163 (7.0)	209 (7.0)	91 (6.4)	375 (5.8)	191 (9.0)	196 (6.5)	104 (8.3)
Graduated from high school	2,900 (26.3)	964 (32.0)	1,763 (23.9)	706 (32.8)	2,852 (25.3)	1,012 (35.2)	1,708 (24.0)	761 (32.2)
Some college	3,493 (31.7)	1,052 (34.3)	2,296 (31.4)	693 (30.6)	3,478 (32.2)	1,067 (32.3)	2,219 (31.2)	770 (31.5)
College graduate	6,342 (35.6)	1,352 (26.7)	4,120 (37.6)	979 (30.2)	6,362 (36.6)	1,332 (23.4)	4,112 (38.3)	987 (28.0)
**Rural residence**								
No	8,718 (82.8)	2,150 (77.5)	5,523 (83.3)	1,438 (76.1)	8,735 (83.5)	2,133 (74.8)	5,406 (83.3)	1,555 (76.3)
Yes	4,420 (17.2)	1,381 (22.5)	2,865 (16.7)	1,031 (23.9)	4,332 (16.5)	1,469 (25.2)	2,829 (16.7)	1,067 (23.7)

Abbreviations: BRFSS, Behavioral Risk Factor Surveillance System; Pap, Papanicolaou.

a Percentages were calculated as column percentages, except for the category for disability, which were calculated as row percentages. All percentages were weighted by using the BRFSS dataset methodology, accounting for the complex survey design of BRFSS, which includes stratification (_ststr), clustering (_psu), and weight (_llcpwt) variables.

In 2018 and 2020, more than 95% of women with health insurance coverage were current with both mammograms and Pap tests. In contrast, among women without health insurance coverage, 3.9% (2018) and 3.4% (2020) were up to date on mammograms and 4.3% (2018) and 4.1% (2020) were up to date on Pap tests. In 2018, by annual household income, women with incomes of $75,000 or more had the highest rates of being up to date on both mammograms (47.7%) and Pap tests (48.0%). Similarly, in 2020, this income bracket had the highest rates (48.6% for mammograms and 49.3% for Pap tests). Married women had consistently higher rates of being up to date on both tests in both years (2018: 69.6% for mammograms, 70.0% for Pap tests; 2020: 70.0% for mammograms, 70.2% for Pap tests). College graduates had the highest rates of being up to date on both mammograms (2018: 35.6%, 2020: 37.6%) and Pap tests (2018: 36.6%, 2020: 38.3%). Additionally, urban residents had higher rates than their rural counterparts in both years for mammograms (2018: 82.8%, 2020: 83.3%) and Pap tests (2018: 83.5%, 2020: 83.3%).

### Adjusted model: independent comparison of mammogram and Pap test screening rates based on SDOH and disability status before (2018) vs during COVID-19 (2020)

**Figure 1:**
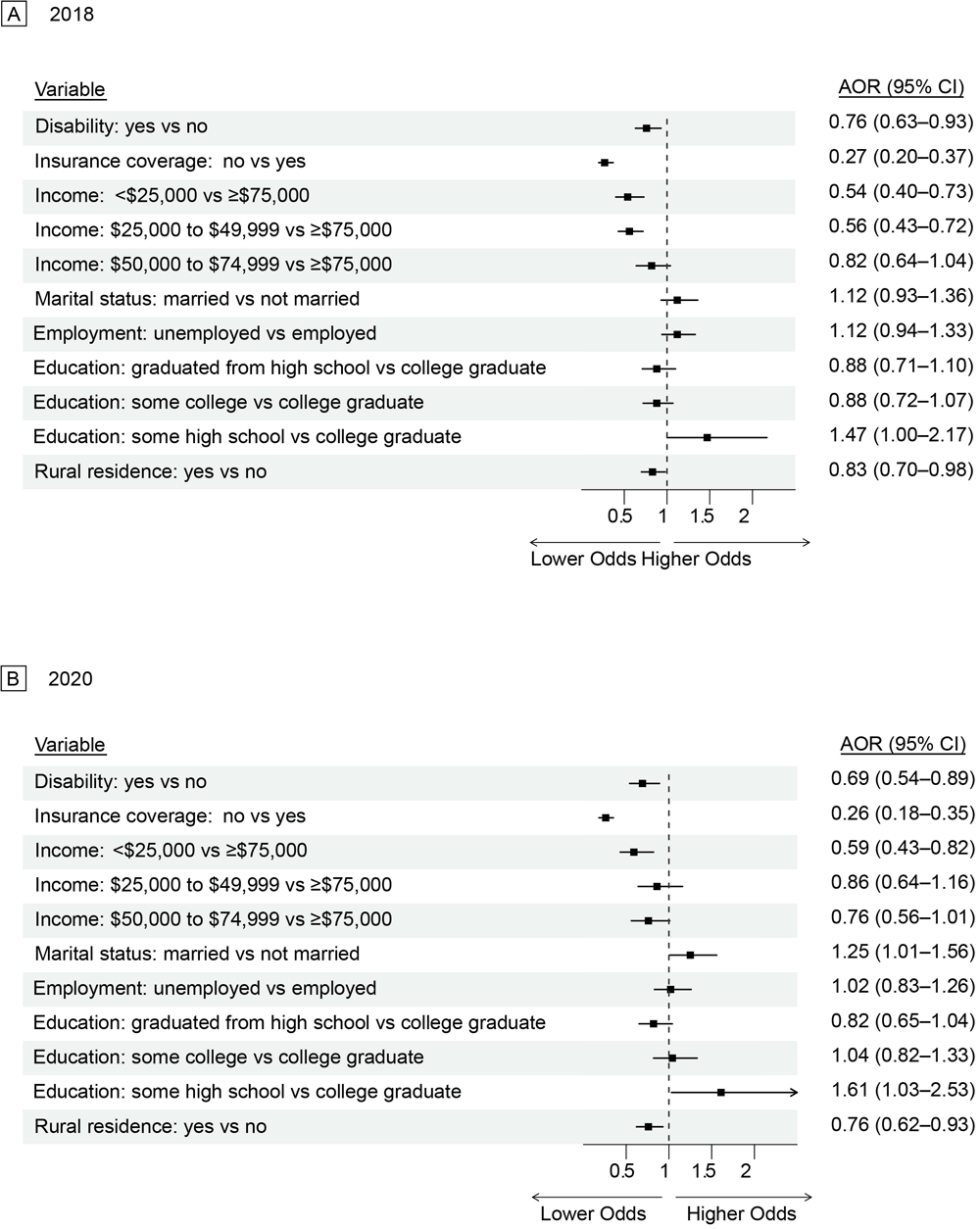
Adjusted odds of being up to date on mammogram screening in A) 2018 and B) 2020 by social determinants of health among all women eligible for screening, Behavioral Risk Factor Surveillance System, 2018 and 2020.

**Figure 2:**
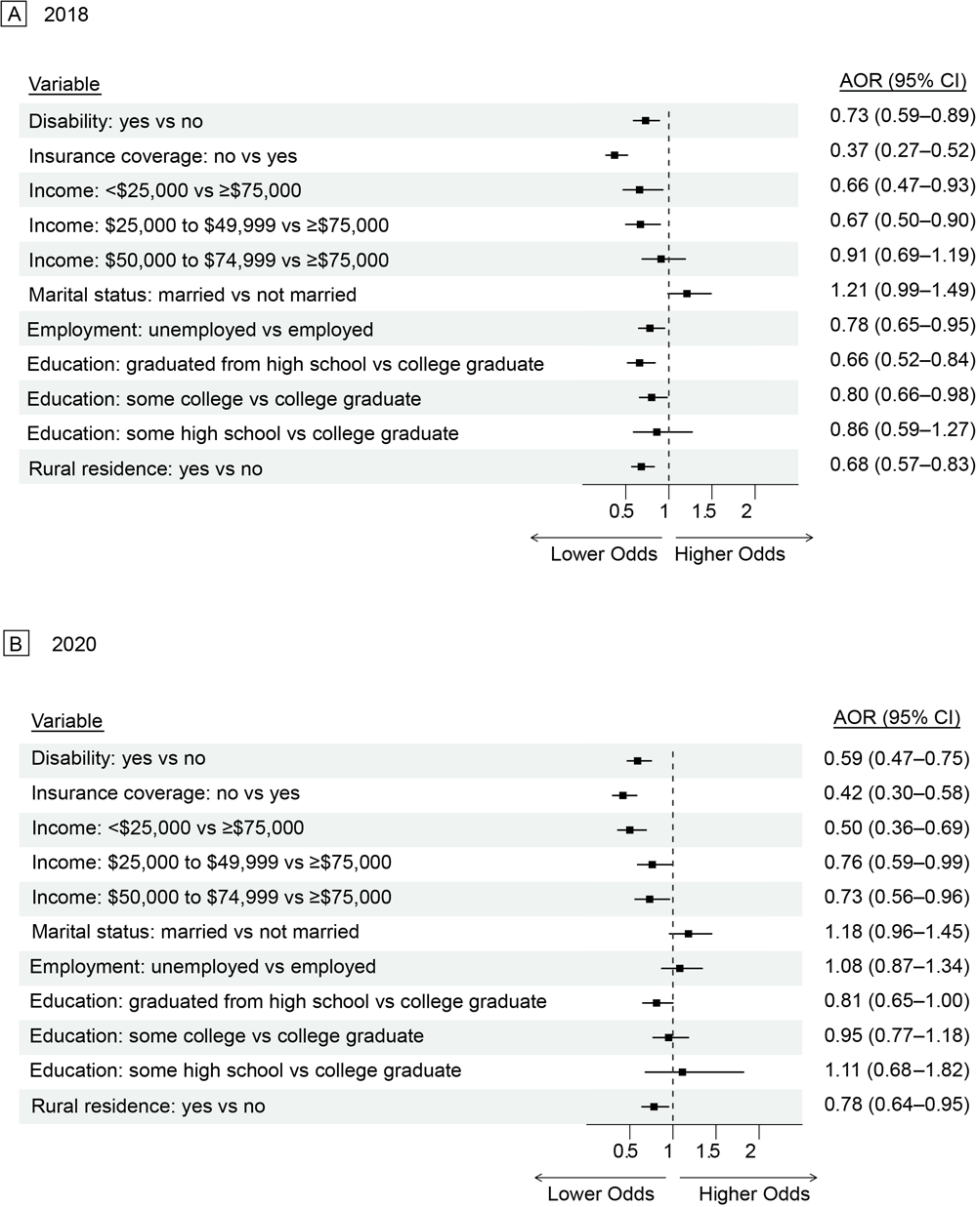
Adjusted odds of being up to date on Pap test screening in A) 2018 and B) 2020 by social determinants of health among all women eligible for screening, Behavioral Risk Factor Surveillance System, 2018 and 2020.

In 2018, women with disabilities had lower odds than women without disabilities of being up to date on mammograms (AOR = 0.76; 95% CI, 0.63–0.93) and Pap tests (AOR = 0.73; 95% CI, 0.59–0.89). In 2020, these odds decreased to 0.69 (95% CI, 0.54–0.89) for mammograms and 0.59 (95% CI, 0.47–0.75) for Pap tests (Figure 1 and Figure 2). Women without health insurance coverage in 2018 had odds of 0.27 (95% CI, 0.20–0.37) for mammograms and 0.37 (95% CI, 0.27–0.52) for Pap tests, compared with women with health insurance coverage. In 2020, these odds changed to 0.26 (95% CI, 0.18–0.35) for mammogram and 0.42 (95% CI, 0.30–0.58) for Pap tests. In 2018, women with an annual household income of less than $25,000, compared with women with an annual household income of $75,000 or more, had an adjusted odds of 0.54 (95% CI, 0.40–0.73) for mammograms and 0.59 (95% CI, 0.43–0.82) for Pap tests. In 2020, these odds were 0.59 (95% CI, 0.43–0.82) for mammograms and 0.50 (95% CI, 0.36–0.69) for Pap tests. In 2018, married women, compared with women who were not married, had an adjusted odds of 1.12 (95% CI, 0.93–1.36) for mammograms and 1.21 (95% CI, 0.99–1.49) for Pap tests. In 2020, these odds changed to 1.25 (95% CI, 1.01–1.56) for mammograms and 1.18 (95% CI, 0.96–1.45) for Pap tests. Among rural residents, compared with urban residents, the adjusted odds in 2018 were 0.83 (95% CI, 0.70–0.98) for mammograms and 0.76 (95% CI, 0.62–0.93) for Pap tests. In 2020, these odds were 0.76 (95% CI, 0.62–0.93) for mammograms and 0.78 (95% CI, 0.64–0.95) for Pap tests. In 2018, women with some high school education, compared with women who were college graduates, had an adjusted odds of 1.47 (95% CI, 1.00–2.17) for mammograms and 1.61 (95% CI, 1.03–2.53) for Pap tests. These odds changed in 2020 to 1.61 (95% CI, 1.03–2.53) for mammograms and 1.11 (95% CI, 0.68–1.82) for Pap tests. Additionally, in 2018, unemployed women had significantly lower odds than employed women (AOR = 0.78; 95% CI, 0.65–0.95) of being up to date on Pap tests; in 2020, the AOR for Pap tests became nonsignificant (AOR = 1.08; 95% CI, 0.87–1.34).

**Table:** 

Variable	Adjusted odds ratio (95% CI)	
	2018	2020
**Disability**		
Yes	0.76 (0.63–0.93)	0.69 (0.54–0.89)
No	1 [Reference]	1 [Reference]
**Insurance coverage**		
No	0.27 (0.20–0.37)	0.26 (0.18–0.35)
Yes	1 [Reference]	1 [Reference]
**Annual household income, $**		
<25,000	0.54 (0.40–0.73)	0.59 (0.43–0.82)
25,000–49,999	0.56 (0.43–0.72)	0.86 (0.64–1.16)
50,000–74,999	0.82 (0.64–1.04)	0.76 (0.56–1.01)
>75,000	1 [Reference]	1 [Reference]
**Marital status**		
Married	1.12 (0.93–1.36)	1.25 (1.01–1.56)
Not married	1 [Reference]	1 [Reference]
**Employment**		
Unemployed	1.12 (0.94–1.33)	1.02 (0.83–1.26)
Employed	1 [Reference]	1 [Reference]
**Education**		
Some high school	1.47 (1.00–2.17)	1.61 (1.03–2.53)
Graduated from high school	0.88 (0.71–1.10)	0.82 (0.65–1.04)
Some college	0.88 (0.72–1.07)	1.04 (0.82–1.33)
College graduate	1 [Reference]	1 [Reference]
**Rural residence**		
Yes	0.83 (0.70–0.98)	0.76 (0.62–0.93)
No	1 [Reference]	1 [Reference]

**Table:** 

Variable	Adjusted odds ratio (95% CI)	
	2018	2020
**Disability**		
Yes	0.73 (0.59–0.89)	0.59 (0.47–0.75)
No	1 [Reference]	1 [Reference]
**Insurance coverage**		
No	0.37 (0.27–0.52)	0.42 (0.30–0.58)
Yes	1 [Reference]	1 [Reference]
**Annual household income, $**		
<25,000	0.66 (0.47–0.93)	0.50 (0.36–0.69)
25,000–49,999	0.67 (0.50–0.90)	0.76 (0.58–0.99)
50,000–74,999	0.91 (0.69–1.19)	0.73 (0.56–0.96)
>75,000	1 [Reference]	1 [Reference]
**Marital status**		
Married	1.21 (0.99–1.49)	1.18 (0.96–1.45)
Not married	1 [Reference]	1 [Reference]
**Employment**		
Unemployed	0.78 (0.65–0.95)	1.08 (0.87–1.34)
Employed	1 [Reference]	1 [Reference]
**Education**		
Some high school	0.86 (0.59–1.27)	1.11 (0.68–1.82)
Graduated from high school	0.66 (0.52–0.84)	0.81 (0.65–1.00)
Some college	0.80 (0.66–0.98)	0.95 (0.77–1.18)
College graduate	1 [Reference]	1 [Reference]
**Residence**		
Rural	0.68 (0.57–0.83)	0.78 (0.64–0.95)
Urban	1 [Reference]	1 [Reference]

### Adjusted model: analysis of SDOH and race and ethnicity among women with disabilities

In 2018, among women with disabilities, the likelihood of being up to date with mammograms was higher among Hispanic (AOR = 2.42; 95% CI, 1.37–4.26) and non-Hispanic Black women (AOR = 2.20; 95% CI, 1.27–3.83) than non-Hispanic White women (Table 2). Income disparities were evident: women with an annual household income of $25,000 to $49,999 had lower odds than women with an annual household income of $75,000 or more of being up to date with mammograms (AOR = 0.47; 95% CI, 0.29–0.74). Compared with non-Hispanic White women with disabilities, Hispanic (AOR = 2.08; 95% CI, 1.16–3.74) and non-Hispanic Black (AOR = 2.04; 95% CI, 1.08–3.87) women with disabilities were more likely to have Pap tests. Women with an annual household income of less than $25,000 had lower odds than women with an annual household income of $75,000 or more of being up to date with Pap tests (AOR = 0.54; 95% CI, 0.31–0.93). In 2020, non-Hispanic Black women had higher odds for mammograms (AOR = 2.70; 95% CI, 1.40–5.21) and Pap tests (AOR = 2.15; 95% CI, 1.19–3.87) than they did in 2018.

**Table 2:** Adjusted Odds^a^ of Being Up to Date on Mammogram and Pap Test by SDOH and Race and Ethnicity Among Women With Disabilities, Behavioral Risk Factor Surveillance System, 2018 and 2020

Variable	Mammogram, AOR (95% CI)		Pap test, AOR (95% CI)	
	2018	2020	2018	2020
**Race and ethnicity**				
Hispanic	2.42 (1.37–4.26)	1.43 (0.51–4.01)	2.08 (1.16–3.74)	2.25 (0.74–6.83)
Non-Hispanic Black	2.20 (1.27–3.83)	2.70 (1.40–5.21)	2.04 (1.08–3.87)	2.15 (1.19–3.87)
Non-Hispanic White	1 [Reference]	1 [Reference]	1 [Reference]	1 [Reference]
**Health insurance coverage**				
No	0.24 (0.15–0.37)	0.27 (0.14–0.52)	0.34 (0.21–0.55)	0.49 (0.26–0.94)
Yes	1 [Reference]	1 [Reference]	1 [Reference]	1 [Reference]
**Annual household income, $**				
<25,000	0.64 (0.38–1.05)	0.67 (0.36–1.24)	0.54 (0.31–0.93)	0.63 (0.34–1.17)
25,000–49,999	0.47 (0.29–0.74)	0.93 (0.49–1.77)	0.53 (0.33–0.83)	0.83 (0.46–1.52)
50,000–74,999	0.88 (0.50–1.54)	1.23 (0.62–2.40)	0.74 (0.41–1.35)	0.90 (0.43–1.88)
>75,000	1 [Reference]	1 [Reference]	1 [Reference]	1 [Reference]
**Marital status**				
Married	1.39 (1.01–1.91)	1.48 (0.96–2.29)	1.32 (0.91–1.92)	1.30 (0.86–1.98)
Not married	1 [Reference]	1 [Reference]	1 [Reference]	1 [Reference]
**Employment**				
Unemployed	1.04 (0.74–1.45)	1.33 (0.90–1.98)	0.82 (0.59–1.14)	1.14 (0.78–1.68)
Employed	1 [Reference]	1 [Reference]	1 [Reference]	1 [Reference]
**Educational attainment**				
Graduated from high school	0.83 (0.59–1.17)	0.78 (0.45–1.36)	0.74 (0.50–1.09)	0.75 (0.44–1.26)
Some college	0.85 (0.61–1.20)	1.26 (0.75–2.10)	0.86 (0.61–1.20)	0.88 (0.53–1.45)
Some high school	0.93 (0.56–1.55)	2.09 (0.99–4.42)	0.72 (0.45–1.16)	1.10 (0.54–2.24)
College graduate	1 [Reference]	1 [Reference]	1 [Reference]	1 [Reference]
**Residence**				
Rural	0.86 (0.64–1.14)	1.10 (0.72–1.68)	0.82 (0.62–1.10)	0.89 (0.60–1.31)
Urban	1 [Reference]	1 [Reference]	1 [Reference]	1 [Reference]

Abbreviation: AOR, adjusted odds ratio; Pap, Papanicolaou; SDOH, social determinants of health.

a Adjusted for race, annual household income, marital status, employment status, health insurance coverage, education level, and rural/urban residence, taking into account the complex survey design factors such as weighting, stratification, and clustering.

## Discussion

Building on existing evidence of how disability status and SDOH influence preventive screening behaviors, our study offers a novel perspective by examining these factors during the COVID-19 pandemic. By analyzing SDOH and disability separately, we aimed to shed light on the unique influence of each on access to preventive care and health outcomes. The pandemic likely heightened or introduced new barriers to use of health care services. Our adjusted models underscored the intricate relationships and complexities of disability status and SDOH in influencing preventive screening behaviors for breast and cervical cancer during the pandemic.

In 2018, SDOH shaped the screening behaviors of women with disabilities. Those earning below $50,000 had lower odds, compared with those earning $75,000 or more, of receiving a Pap test or mammogram, and married women had higher odds than unmarried women of receiving a mammogram. Regardless of the screening type, health insurance access was critical, and its absence hampered rates of receipt.

During the COVID-19 pandemic in 2020, we found shifts in screening dynamics among racial and ethnic minority groups. Racial differences in rates of receipt for mammograms were more pronounced in 2020 than in 2018: the odds of being up to date with screening among non-Hispanic Black women, compared with non-Hispanic White women, were higher in 2020 than in 2018. Although screening rates might be increasing among racial and ethnic minority groups, addressing the broader disparities in breast and cervical cancer outcomes requires a comprehensive approach that encompasses early detection, equitable access to high-quality care, culturally sensitive health care delivery, and ongoing support throughout the cancer care journey. Meanwhile, disparities in being up to date with screening persisted from 2018 to 2020, but with attenuated intensity. The central role of health insurance coverage also persisted, with lack of insurance consistently associated with reduced odds of screening uptake.

We found that mammogram and Pap test screening rates among women with disabilities declined by 2.5 percentage points (from 72.1% to 69.6% for mammograms) and 3.3 percentage points (from 69.4% to 66.1% for Pap tests), respectively, from 2018 to 2020, indicating an exacerbation of disparities based on disability during COVID-19. The finding that women with disabilities had lower odds than the general population of being up to date on breast and cervical cancer screenings before and during the pandemic corroborates previous findings that highlighted challenges in accessing health care services among people with disabilities (13). Similar patterns of health care underutilization have been reported among people with disabilities across a range of preventive services and medical examinations (21,22). This underutilization may be attributed to various factors, such as physical accessibility, communication barriers, and lack of health care provider expertise in managing patients with disabilities; these factors warrant further research (23). Research on disability and health behaviors underscores the effect of these factors on the engagement of people with disabilities in preventive behaviors (24,25). During the COVID-19 pandemic, these factors were most likely intensified.

Interventions need to be tailored to the unique needs and challenges of people with disabilities, encompassing strategies such as individualized communication, physical adjustments, and specialized health care provider training (23). The design of interventions aimed at promoting mammograms and Pap tests among this group must prioritize the accessibility and adaptability of health care facilities and services, especially during a public health crisis.

We examined the relationship between economic factors and mammograms and Pap tests. Women with higher income and health insurance coverage had higher odds of being up to date with screening. Our findings resonate with recent studies indicating financial constraints and lack of health insurance as barriers to mammogram screening (26). Expanding access to affordable health insurance and reducing out-of-pocket costs for preventive services should be prioritized. 

Studies by Wong et al and Friedman demonstrated that people with disabilities were more financially affected by the pandemic than their counterparts without disabilities. These financial challenges, including job loss and reductions in income, amplified the existing barriers to preventive health care services. More than half of people with disabilities surveyed reported difficulties in paying for usual household expenses during the pandemic (27). Many people relied on credit cards, loans, or borrowing from friends and family to meet their needs (27). Increased financial hardship among people with disabilities, particularly women, could extend to preventive health services such as mammograms and Pap tests (27,28). Women with disabilities, low income, or lost income may forgo these services, potentially leading to late-stage diagnosis and poorer health outcomes. Our findings, in alignment with previous literature, emphasize the necessity to address the economic barriers influencing health-seeking behaviors and the need for inclusive health care strategies during public health emergencies.

Our research provides a nuanced understanding of how marital status and educational attainment influenced screenings during the pandemic. The observed association between marital status and adherence to mammograms and Pap tests highlights the crucial role of SDOH in health behaviors. Although we did not find a significant association between educational attainment and odds of being up to date on mammograms or Pap tests in our adjusted model, higher educational attainment has been shown to positively affect health-seeking behaviors in other studies (29). The discrepancy between our findings and previous findings may suggest that the influence of education may interact with other factors in complex ways, requiring further research. Nevertheless, considering the broader evidence linking educational attainment to health-seeking behaviors, public health initiatives should focus on strategies that can appeal to people with lower education levels or people who lack social support. These interventions could be implemented through community-based interventions or partnerships with educational institutions.

Our research uniquely evaluated health care accessibility and use in the context of rural and urban disparities. We found a significant association between urban residency and adherence to mammogram and Pap test screening: the odds of being up to date with mammograms and Pap tests were lower among rural residents than urban residents. Differences in health care access between urban and rural areas may contribute to disparities in adherence to mammograms and Pap test screening (30). Innovative solutions, such as mobile mammography units and telemedicine consultations, can improve access to screening services in rural and underserved areas (31). Novel approaches, such as mail-in self-sampling for cervical cancer screening, can help address accessibility and acceptability issues in this population (32). An evaluation of health care accessibility and use among disabled people during the COVID-19 pandemic is of paramount importance.

### Limitations

Our study has several limitations. First is the cross-sectional design of the data set. Although our approach allowed us to generate a snapshot of data at 2 points in time, it inherently precluded the ability to infer causality. Second, our reliance on the BRFSS data set, which uses self-reported data, might have introduced recall bias, response bias, or social desirability bias. Although the BRFSS data set is a robust and widely used resource in public health research, the potential discrepancies in self-reported data versus actual behaviors or status cannot be ignored. Third, we did not test whether changes in being up to date on screening from 2018 to 2020 were significant. Future studies using a longitudinal design and validated self-reported data with objective measures may provide more precise findings and elucidate the causal relationships between disability status, SDOH, and cancer screenings during health crises such as the COVID-19 pandemic.

### Conclusion

Our study reaffirms the significance of SDOH in mammogram and Pap test screening behaviors. The effect of disability status, income, health insurance coverage, marital status, educational attainment, and urban or rural residence on screening adherence for breast and cervical cancer during the COVID-19 pandemic has magnified pre-existing health care challenges and disparities. Considering the unique circumstances brought about by the pandemic, it is crucial to design interventions that address the barriers imposed by sociodemographic factors. By enhancing accessibility, affordability, and awareness of screenings, especially among populations who lack access to health care, we could mitigate the detrimental effects of a health care crisis like the pandemic on breast and cervical cancer screening rates. A tailored approach could contribute to reducing disparities and improving breast cancer outcomes.
